# Determination of antiviral action of long non-coding RNA loc107051710 during infectious bursal disease virus infection due to enhancement of interferon production

**DOI:** 10.1080/21505594.2019.1707957

**Published:** 2019-12-28

**Authors:** Xuewei Huang, Yigang Xu, Qingyu Lin, Weilong Guo, Dongfang Zhao, Chunmei Wang, Li Wang, Han Zhou, Yanping Jiang, Wen Cui, Xinyuan Qiao, Yijing Li, Guangpeng Ma, Lijie Tang

**Affiliations:** a College of Veterinary Medicine, Northeast Agricultural University, Harbin, P.R. China; b Agricultural High Technology Department, China Rural Technology Development Center, Beijing China

**Keywords:** Infectious bursal disease virus, lncRNA, loc107051710, *IRF8*, type I interferon, interferon-stimulated gene

## Abstract

The functions and profiles of lncRNAs during infectious bursal disease virus (IBDV) infection have not been determined, yet. The objectives of this study were to determine the antiviral action of loc107051710 lncRNA during IBDV infection by investigating the relationship between loc107051710 and IRF8, Type I IFN, STATs, and ISGs. DF-1 cells were either left untreated as non-infected controls (n = 1) or infected with IBDV (n = 3). RNA sequencing was applied for analysis of mRNAs and lncRNAs expression. Differentially expressed genes were verified by RT-qPCR. Then identification, of 230 significantly different expressed genes (182 mRNAs and 48 lncRNA) by pairwise comparison of the infected and control groups, was carried out. The functions of differentially expressed lncRNAs were investigated by selection of lncRNAs and mRNAs significantly enriched in the aforementioned biological processes and signaling pathways for construction of lncRNA-mRNA co-expression networks. The techniques of gene ontology and Kyoto Encyclopedia of Genes and Genomes pathways were applied. It was suggested that these differentially expressed genes were involved in the interaction between the host and IBDV. Loc107051710 was found to have potential antiviral effects. RT-qPCR and western blot were applied and revealed that loc107051710 was required for induction of IRF8, type I IFN, STAT, and ISG expression, and its knockdown promoted IBDV replication. By fluorescence in situ hybridization, it was found that loc107051710 was translocated from the nucleus to the cytoplasm after infection with IBDV. Overall, loc107051710 promoted the production of IFN-α and IFN-β by regulating IRF8, thereby promoting the antiviral activity of ISGs.

## Introduction

Infectious bursa disease (IBD) is a highly contagious and immunosuppressive disease that affects young chickens. It causes high mortality rates and large economic losses to the poultry industry [1,2]. Infectious bursal disease virus (IBDV) mainly causes the destruction of B-lymphocytes in the bursa of Fabricius, which can lead to severe immunosuppression and secondary infections in infected chickens [3–5]. The virus (IBDV) is non-enveloped with an icosahedral capsid. It belongs to the family *Birnaviridae*. The genome of IBDV is bi-segmented, double-stranded RNA [6].

While the importance of protein encoding genes has long been known, the various types of noncoding RNAs are increasingly receiving attention [7]. Long noncoding RNAs (lncRNAs), which are composed of more than 200 nucleotides, in particular, are involved in apoptosis during organ development and tissue differentiation [8]. They also play regulatory roles in many diseases, particularly, human cancers [9–11], and in the adaptive and innate immune responses [8,12]. Moreover, the lncRNAs have antiviral functions by changing the expression levels of IFNs or ISGs against Theiler’s virus, encephalomyocarditis virus, hepatitis C virus, influenza A virus, and HIV [13–17].

Although DF-1 cells, a continuous line of chicken embryo fibroblasts, have been used to study the transcriptional changes and differentially expressed proteins of host cells in response to IBDV infections [18–24], the role of host lncRNAs in response to IBDV infection is still unknown. Application, of some sophisticated techniques such as techniques of in depth bioinformatics, RNA sequencing, RT-qPCR, Fluorescence in situ hybridization, and western blotting, is expected to provide a theoretical basis for novel preventive and therapeutic strategies against IBDV. The objective of this study was to determine the antiviral action of loc107051710 during IBDV infection in cultured DF-1 cells after investigation of the relationship between the loc107051710 and IRF8, type I IFNs, STATs and ISGs. The antiviral action of loc107051710 was determined by application of RNA-seq for analysis of mRNAs and lncRNAs expression. Next, identification of differentially expressed genes by pairwise was carried out. After that, the functions of differentially expressed lncRNAs were investigated by selection of lncRNAs and mRNAs significantly enriched in the aforementioned biological processes and signaling pathways for construction of lncRNA-mRNA co-expression networks. The techniques of GO and KEGG pathways were applied. Subsequently, RT-qPCR and western blot were applied to investigate the relationship between loc107051710 and IRF8, type I IFNs, STATs, and ISGs expression. Eventually, fluorescence in situ hybridization was applied to determine the location of loc107051710.

## Materials and methods

### Cells and viruses

Chicken embryonic fibroblast DF-1 cells (CRL-12,203, ATCC) were cultured in high-glucose (4.5 g D-glucose/L) Dulbecco’s modified Eagle’s medium (Life Technologies, Grand Island, NY, USA). Then, this medium was supplemented with 10% fetal bovine serum (Invitrogen, Carlsbad, CA, USA), and the culture was incubated at 37°C with 5% CO_2_. IBDV strain CEF94 (College of Veterinary Medicine, Northeast Agricultural University, China) was propagated in DF-1 cells. The tissue culture infectious dose 50 (TCID50) was 10 ^5.25^/0.1 mL. Cells were incubated with IBDV at a multiplicity of infection of 1 (MOI = 1) for 24 h.

### Experimental design and RNA extraction for sequencing

The DF-1 cell cultures were divided into two groups. The first group was infected with IBDV (n = 3). The second group was kept without infection as a control (n = 1). The total RNA of the control (n = 1) and IBDV-infected (n = 3) groups was extracted using TRIzol reagent (Invitrogen, Carlsbad, CA, USA) according to the manufacturer’s instructions. The purity, concentration, and integrity of the total RNA were analyzed using an Agilent Bioanalyzer 2100 (Agilent Technologies, Santa Clara, CA, USA). Samples with an RNA integrity number (RIN) value > 9.7 and an optical density 260:280 ratio > 2.0 were used for library construction and deep sequencing. RNA-seq was performed by Novel Bioinformatics Co., Ltd. (Shanghai, China) using the Illumina XTen platform.

### Quality control of RNA readings

The raw readings were preprocessed with custom Perl scripts to ensure the high quality of RNA for subsequent analyzes. Adapter-polluted readings, low-quality readings (in which more than 15% of bases had a Phred score ≤ 19, or more than 5% were undetermined, N), and readings matching rRNA were removed.

### Alignment, mapping of RNA-seq to reference genome and transcriptome assembly

Using TopHat version 2.0.12 [25] the RNA-seq readings were aligned and mapped to the reference genome obtained from NCBI Gallus_gallus-5.0 (ftp://ftp.ncbi.nlm.nih.gov/genomes). Using the Cufflinks 2.2.1 program [26], the transcriptome of each sample was assembled independently with the help of the reference annotation-based transcript assembly technique.

### Selection of lncRNAs

Based on the assembly results, some transcripts were removed such as transcripts with readings of 0. For selection of lncRNAs, the following transcripts were removed: Transcripts which were shorter than 200 nucleotides and having less than two exons, transcripts which were only present in one sample, and transcripts which were encoding a protein family or were a known mRNA transcript. The Coding-Non-Coding Index (CNCI) [27], Coding Potential Calculator (CPC) [28], and Coding-Potential Assessment Tool (CPAT) [29] were used to evaluate the coding potential of the transcripts. Default parameters were used with all the software. After filtering, transcripts without coding potential were considered candidate lncRNAs.

### Quantification of gene expression

The read count for each gene in each sample was determined by HTSeq v0.6.0 [30], and the number of fragments per kilobase of transcript sequence per million base pairs sequenced [31] was calculated to measure the expression levels of both mRNAs and lncRNAs in each sample. DESeq v1.16.0 [32] was used for the differential expression analysis of two groups. Genes, with a *p* value <0.05 and a fold change >1.5, were considered differentially expressed in the two groups.

### Determination of significantly enriched biological functions and pathways in mRNAs differentially expressed in IBDV infected DF-1 cells and control DF-1 cells

Using fisher.test and p.adjust routines, 2 analyses were performed with custom R scripts. These 2 analyses were gene ontology (GO) and Kyoto encyclopedia of genes and genomes (KEGG) pathway enrichment. These analyses were used to determine biological functions and pathways significantly enriched in mRNAs differentially expressed in IBDV infected DF-1 cells and control DF-1 cells. GO categories and KEGG pathways with *p* values <0.05 were considered significantly enriched.

### Protein-protein and lncRNA-mRNA co-expression network analyses

We constructed gene co-expression networks to identify the interactions among differentially expressed genes. The gene co-expression networks were built using the normalized signal intensity of specific expression genes. For each pair of genes, the Pearson correlation coefficient was calculated, and significantly correlated pairs were selected to construct the networks. In network analysis, degree centrality* was the simplest and most important measure of the relative importance of a gene within a network. Moreover, to analyze certain properties of the networks, k-cores**, from graph theory, were introduced for simplifying graph topology analysis. In the present study, the purpose of the network structure analysis was to locate genes in one network. When analyzing the different networks, genes with the largest degree of difference between the two classes were selected. (Note: *Degree centrality is defined as the number of links connecting one node to other nodes; **A k-core of a network is a subnetwork in which all nodes are connected to at least k other genes in the subnetwork. Accordingly, a k-core of a protein-protein interaction network contains cohesive groups of proteins).

### Gene silencing of loc107051710

A specific siRNA for loc107051710 was designed by Genepharma Co., Ltd. (Shanghai, China). The sequences of the specific and control siRNA are listed in Table 1. In total, 5 × 10^5^ DF-1 cells were seeded in 6-well plates, and when they reached 50–60% confluence, they were transfected with 100 nM negative siCont or siloc107051710 using Lipofectamine 2000 (Invitrogen, Carlsbad, CA, USA) according to the manufacturer’s instructions. After transfection for 24 h, the cells were infected for 24 h with IBDV (MOI = 1).

**Table 1.** SiRNA and probe sequence of loc107051710.

Sequence name	Primer sequence (5ʹ-3ʹ)
Loc107051710-sense	GCAAGACAGCAUUUGGUAUTT
Loc107051710-antisense	AUACCAAAUGCUGUCUUGCTT
Negative control-sense	UUCUCCGAACGUGUCACGUTT
Negative control-antisense	ACGUGACACGUUCGGAGAATT
Loc107051710-antisense probe	CCAGTATGGAAGAAGTTGAAAATG
Loc107051710-sense probe	TGTTGGTCCTTTGTCTGTGATCAG

### Quantitative reverse transcription PCR (Rt-qPCR)

Total RNA was extracted from control and IBDV-infected groups using TRIzol according to procedures previously described and reverse-transcribed to cDNA using M-MLV Reverse Transcriptase (Promega, Madison, WI, USA) according to the manufacturer’s procedures. Twenty-four differentially expressed genes were selected to confirm the accuracy of RNA-seq by RT-qPCR, including 12 lncRNAs and 12 mRNAs. Moreover, RT-qPCR was applied for detection of the expression of type I IFNs, STATs and ISGs. Primers were designed using oligo6 software (Tables 2–4). For RT-qPCR, the ABI 7500 Real-Time PCR system (Applied Biosystems, Carlsbad, CA, USA) and SYBR Green Master (TaKaRa, Dalian, China) were used; the reactions were performed in a final volume of 20 μL. The *GAPDH* gene was used as an internal control. The relative fold change was calculated by the 2^−ΔΔCt^ method [33]. Experiments were repeated three times.

**Table 2.** RT-qPCR primers used for verification of mRNA results.

Gene	Primer sequence (5ʹ-3ʹ)	Accession number
GAPDH	F-TGACCACTGTCCATGCCATC	NM_204305
	R-CAGCAGCCTTCACTACCCTC	
IFI6	F-TCAGGCTTTACCAGCAGTGG	NM_001001296
	R-TGCCACCCATTGAGATAGACTG	
IRF1	F-TAAGCATGGCTGGGACATGG	XM_015294015
	R-GGAGCATCCTGTACACTCGG	
IFIT5	F-CCCTCTCAAGCTGAAGCACT	NM_001320422
	R-TGAACAGACAAGCAAACGCA	
TLR3	F-ACATTTGTAACACCCCGCCT	FJ915471
	R-CCCGGTAGTCTGTCAAGCTC	
RSAD2	F-GCAGTGCAACTACAAGTGTG	NM_001318443
	R-GAAATGGTTCTCCTCCTGAG	
OASL	F-GTCAGCATCACCAGTCCG	XM_015293006
	R-CAGTGCGTCGTAAGCAGG	
DUSP18	F-GTGCAAGGAGGAAGGAGGA	XM_415295.6
	R-GACGGTGGTGATGTGGTTG	
BEST3	F-GGAACGATTCTTCTGCACG	NM_001199669.1
	R-GGAAGAACACCGAGTTCTCAC	
ZNF414	F-GAAGCACTACGCCTGCTCCAG	XM_015299849.2
	R-AAGGTCTCTGTGCAGCCCAGC	
PLVAP	F-AACCTGACACGAACCCTCAAC	XM_003643487.4
	R-CCTTCTGATGTCCTCTAGCCTG	
CCLi10	F-CTCTGCTCCTCGGCTGTG	FR874034.1
	R-AGGCAATGAGGTTGCGTG	
TNS4	F-TGTCCCACCCAGCCTCTC	XM_015299654.2
	R-GGAGGGGTACCCCACCTG

**Table 3.** RT-qPCR primers used for verification of lncRNA results.

Gene	Primer sequence (5ʹ-3ʹ)	Accession number
loc107051710	F-GAGGAATTGAGGAGTGAGTG	XR_001463517
	R-ATGTCCTGGTCCTCGTTC	
loc107052218	F-GAGTCCCAGGACAGCATG	XR_001464528
	R-AAGTCTGGCTGGTGGCTG	
loc107052259	F-CAATCACAGAGCAGTGCG	XR_001464642.2
	R-CTGCATTCCAGGGTGAGG	
loc107050176	F-GGGAGCTGCGAAGGATGAGT	XR_001462440
	R-CCAGCGCTCCAAAGCTTCAC	
loc107053553	F-CAGCAGTGCTCCCAGCAG	XR_001466914
	R-ACCTGGTGAGGGCAGGCT	
loc107052689	F-ACCTCCTCCTTGTGGAAG	XR_001465330
	R-ACCTGCAGTTTCACAGGG	
loc107053273	F-TGTTGGGTGTACGTCCTTC	XR_003074877.1
	R-ATCGGTGGTATCTGACAGC	
loc107054529	F-CCATGCAAGCTGTGTGAG	XR_001468879.1
	R-CAGTCCATCTGCATGCAC	
loc107052150	F-CGAACTCCAGAGGAACTG	XR_001464380.2
	R-TCAAGCCCATAGATGCAG	
loc107054795	F-GGCCTATTCTATGCAGCATG	XR_003071209.1
	R-CAGAAGAAGGGCTCATGAAG	
loc107052781	F-AGGCGAATAGCAGAGGTAC	XR_001465506.2
	R-GCTCAGAGCTCCAGTAAAG	
loc107055337	F-CAAGATCACCAGGTGCAAC	XR_001470403.2
	R-CCTGTTGGGTCATGACATG

**Table 4.** RT-qPCR primers sequences.

Gene	Primer sequence (5ʹ-3ʹ)	Accession number
OAS	F-CCACATCCTCGCCATCATCG	NM_205041
	R-GTCTGTCCCGGCTGTCCTTG	
Mx1	F-GGTGTCATTACTCGCTGT	GU256272
	R-CTTTCTTCACCTCTGATGC	
PKR	F-GGCACCAGAACAGTTTGG	AB125660
	R-GTTCAGTGGAAGGTCACC	
STAT1	F-CGATGACAGCTTTCCTATGG	XM_015289392
	R-GCAGGTCATGGAATAGCAC	
STAT2	F-CCCGAGAACTTTGCCAACC	XM_015300285
	R-GATCTCCTGCTCCTGGCTG	
IRF8	F-CCGTCAGAAGCAGATCACCA	XM_015292553
	R-TTGCTTGGCCTGTCCTTGTA	
IFN-α	GACATGGCTCCCACACTACC	GU119896
	AGGCGCTGTAATCGTTGTCT	
IFN-β	CCTCAACCAGATCCAGCATT	GU119897
	GGATGAGGCTGTGAGAGGAG	
IBDV-VP2	F- GTCAAGCACACTTCCTGGTG	AF194428
	R- GTCGTTGATGTTGGCTGTTG	

### Western blotting

Total proteins were extracted, subjected to 15% SDS-PAGE and transferred to PVDF membranes. After blocking with 5% skim milk overnight, the PVDF membranes were incubated at 37°C for 1 h with anti-chicken IFN-α and IFN-β antibodies (1:1000; Cloud-Clone Corp, Houston, TX, USA) and anti-IBDV VP2 polyclonal antibodies (1:500) prepared in our laboratory. Subsequently, the samples were incubated with horseradish peroxidase-conjugated goat anti-rabbit IgG secondary antibodies (1:3000; Cloud-Clone Corp., Houston, TX, USA) at 37°C for 40 min, and the immunoblots were visualized with an enhanced chemiluminescence system (Cheml Scope5300; Clinx Science Instruments, Shanghai, China). β-actin was used as an internal control.

### Fluorescence in situ hybridization (FISH)

Using FISH technique, loc107051710 was detected on DF-1 cells. The biotin-labeled antisense and sense probes of loc107051710 were synthesized by RiboBio (Guangzhou, China) and the sequences are listed in Table 1. The FISH assay was performed using a Fluorescent In Situ Hybridization Kit (RiboBio, Guangzhou, China) according to the manufacturer’s procedures. In brief, DF-1 cells were fixed with 4% polyoxymethylene. After washing with PBS, the cells were treated with 0.5% Triton X-100 at 4°C for 5 min. Next, the cells were treated with pre-hybridization lncRNA FISH probe mix at 37°C for 30 min. Hybridization was performed by the adding antisense and sense loc107051710 FISH probe mix (10 mM ATP, 10 mM CTP, 10 mM GTP, 6.5 mM UTP and 3.5 mM Biotin-16-UTP), followed by incubation at 37°C overnight. After washing with 4×, 2×, and 1× SSC, the cells were stained with DAPI, washed three times with PBS, and visualized by laser scanning confocal microscopy.

### Statistical analyses

The data were analyzed using GraphPad Prism5 software. One-way ANOVA was used to determine significance. *P* values <0.05 were considered statistically significant.

## Results

### Overview of lncRNA and mRNA expression profiles

In total, 361 100 380 raw reads were obtained from the infected and control DF-1 cells (NCBI SRA Run Selector, accession number SRP145165). After quality control, 308 057 830 clean reads (readings after removal of Adapter-polluted readings, low-quality readings and readings matching rRNA), were obtained, and the percentage of clean reads ranged from 83.74% to 87.27%. The percentage of clean reads with a Phred quality value of more than 30 ranged from 95.03% to 95.54% (Table 5). Overall, 86-87% of the clean reads aligned with the chicken reference genome (Table 6).

**Table 5.** Data quality of lncRNA and mRNA profiles.

Sample name	Raw reads	Clean reads	Clean reads rate (%)	Q30 (%)
JD01c	81,795,032	68,492,856	83.74	95.03
JD02c	83,442,316	69,873,950	83.74	95.07
JD03c	93,319,420	81,440,754	87.27	95.54
CK01c	102,543,612	88,250,270	86.06	95.22

**Table 6.** Clean reads compared with the reference genome.

	Samples			
	CK01C	JD03C	JD01C	JD02C
Total Reads	88,129,420	81,326,852	68,412,444	69,781,248
Mapped Reads	76,989,566	70,851,841	58,920,072	60,073,583
Mapping Rate (%)	0.87	0.87	0.86	0.86
UnMapped Reads	11,139,854	10,475,011	9,492,372	9,707,665
MultiMap Reads	2,064,678	1,904,761	1,616,128	1,643,890
MultiMap Rate (%)	0.02	0.02	0.02	0.02

### Identification of differentially expressed lncRNAs and mRNAs

**Figure 1.**
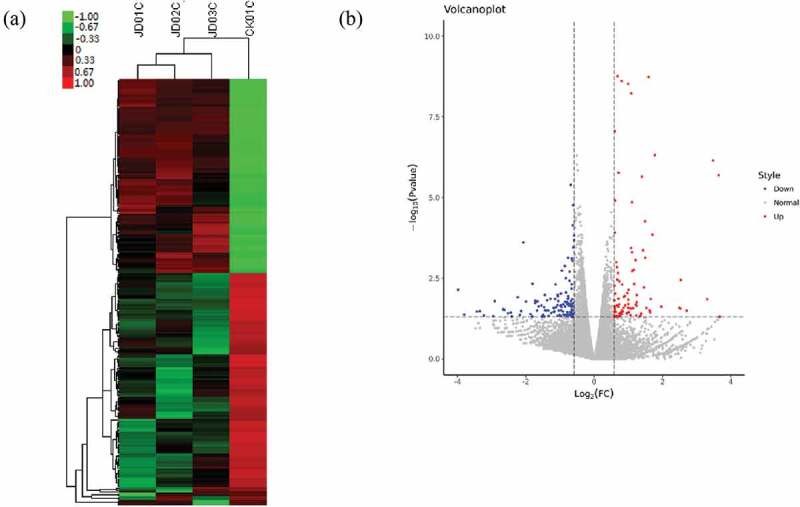
RNA-seq analyses analysis of IBDV-infected DF-1 cells. (a) Heatmap of differentially expressed mRNAs and lncRNAs. Red represents a higher transcription, and green represents a lower transcription. (b) Volcano plots of differentially expressed mRNAs and lncRNAs. The red, blue, and gray dots represent significantly upregulated genes, significantly downregulated genes, and unchanged genes, respectively.

To analyze the differential expression of mRNAs and lncRNAs between the control and IBDV-infected groups, heat maps (Figure 1(a)) and volcano plots (Figure 1(b)) were generated. Given the criteria of *p* value <0.05 and fold change >1.5, 91 mRNAs were upregulated and 91 were downregulated (Table 7, supplementary materials 1) . Furthermore, 13 lncRNAs were upregulated, and 35 lncRNAs were downregulated in IBDV- infected DF-1 cells (Table 7, supplementary materials 2).

**Table 7.** The number of differentially expressed mRNA and lncRNA in IBDV treated group.

Comparison	Up-regulated	Down-regulated
mRNA	91	91
LncRNA	13	35

### Functional annotation of differentially expressed mRNAs

**Figure 2.**
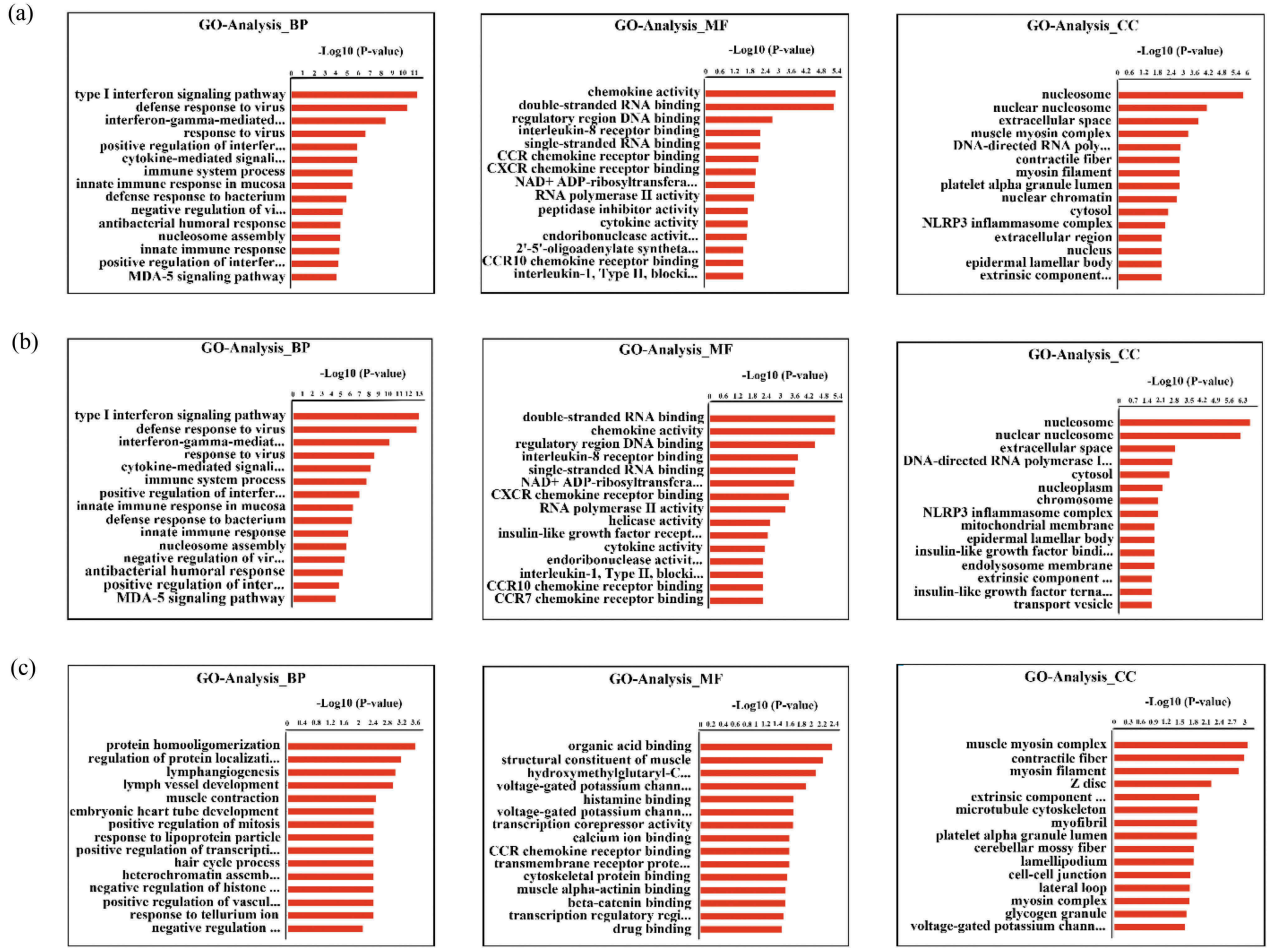
GO enrichment of mRNAs differentially expressed in the infected and control groups with a fold change >1.5 and a *p* value <0.05. (a) Biological process enrichment analysis of all differentially expressed mRNAs. (b) Biological process enrichment analysis of upregulated mRNAs. (c) Biological process enrichment analysis of downregulated mRNAs. The Y-axis shows the pathways, and the X-axis shows the negative logarithm of the *p* value (-log *P*).

**Figure 3.**
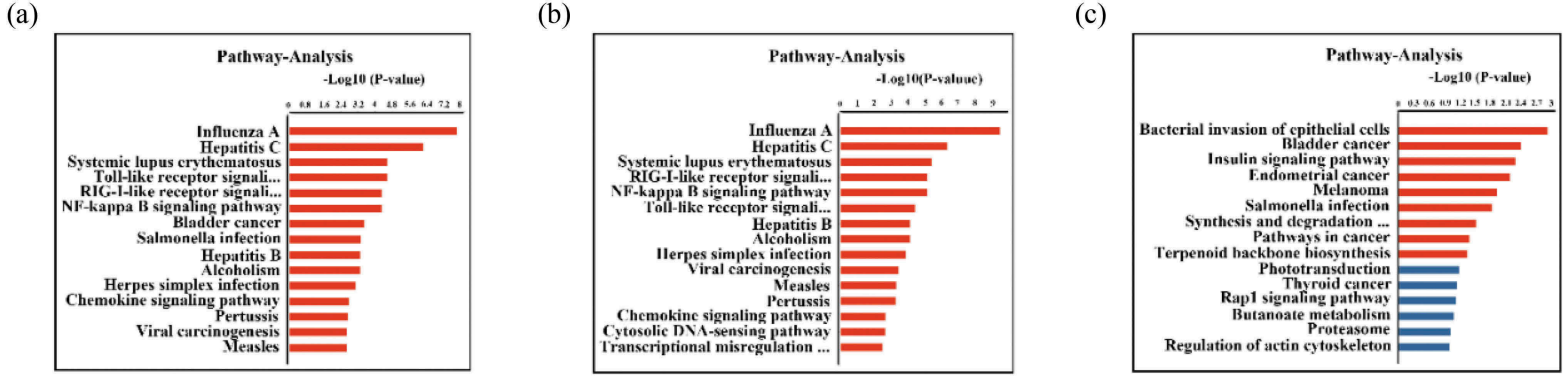
KEGG pathway enrichment of differentially expressed mRNAs with a fold change >1.5 and a *p* value <0.05. (a) Pathway enrichment analysis of all differentially expressed mRNAs. (b) Pathway enrichment analysis of upregulated mRNAs. (c) Pathway enrichment analysis of downregulated mRNAs. The Y-axis shows the pathways, and the X-axis shows the negative logarithm of the *p* value (-log *P*).

In total, 182 differentially expressed mRNAs were subjected to GO and KEGG pathway enrichment analyses (Figures 2 and 3) to obtain a better understanding of the potential roles of host factors in IBDV infection. We found that the GO terms were significantly enriched in differentially expressed mRNAs, as well as for just upregulated mRNAs, were mainly involved in biological processes and pathways that may be related to the replication of IBDV, including the type I IFN signaling pathway, defense response to virus, IFN-γ-mediated signaling pathway, and positive regulation of the type I IFN production signaling pathway (Figure 2 and supplementary materials 3). Moreover, KEGG analysis showed that differentially expressed genes were involved in the RIG-I-like receptor, NF-κB, Toll-like receptor, influenza A, and hepatitis C signaling pathways (Figure 3, supplementary materials 4).

### Protein-protein interaction network analysis

**Figure 4.**
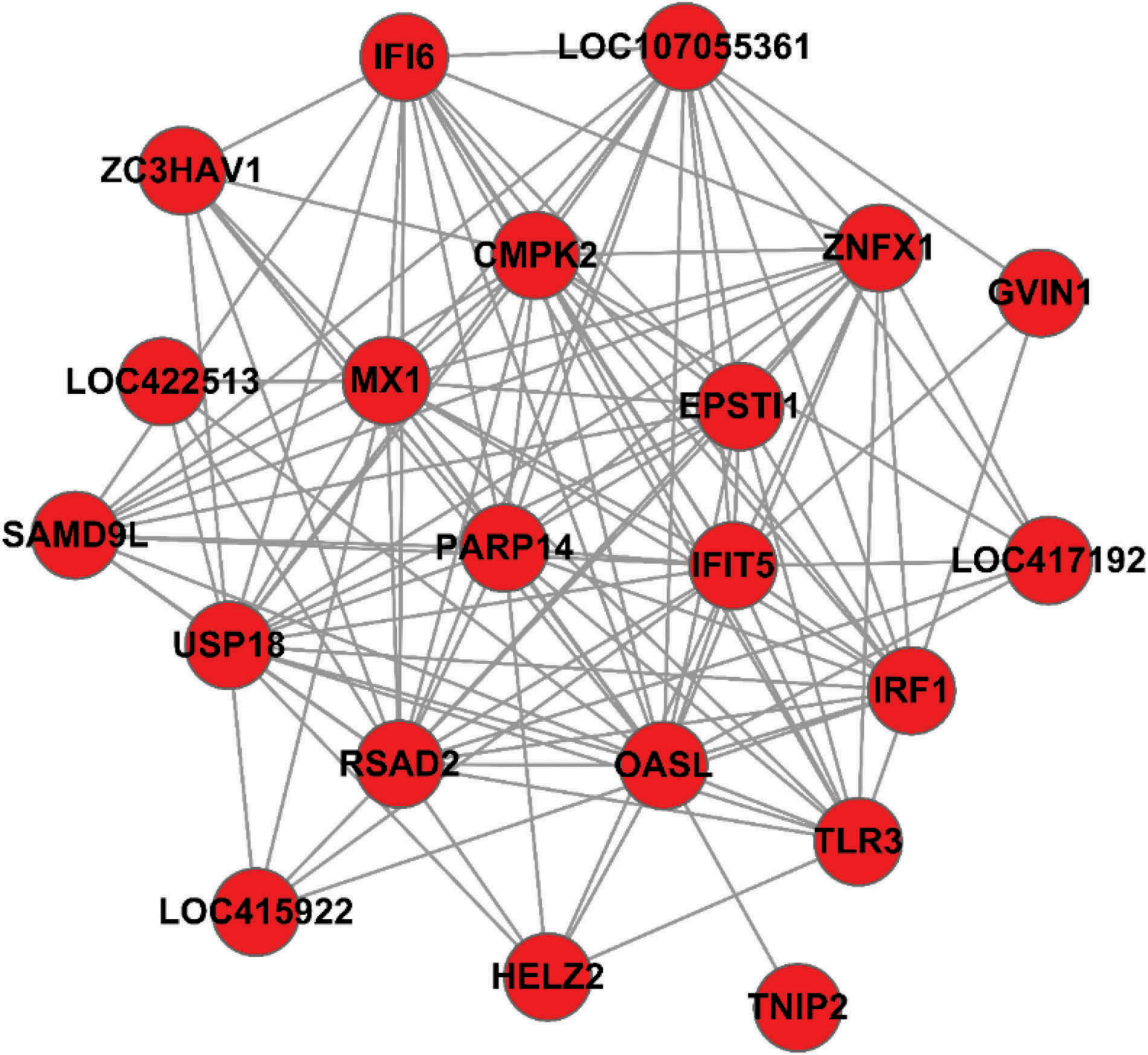
Protein-protein interaction network. A node indicates a differentially expressed mRNA. A node linked to more nodes indicates that it is more important relative to other nodes in this network.

mRNA-mRNA functional interaction network analysis demonstrated that *IFIT5, OASL, USP18, TLR3, IRF1, IFI6, SAMD9L, EPSTI1*, and several other mRNAs were at the core of the network (Figure 4). Most of these mRNAs were upregulated and significantly enriched in the type I IFN signaling pathway and the RIG-I, NF-κB, and Toll-like receptor signaling pathways.

### lncRNA-mRNA co-expression network analysis

**Figure 5.**
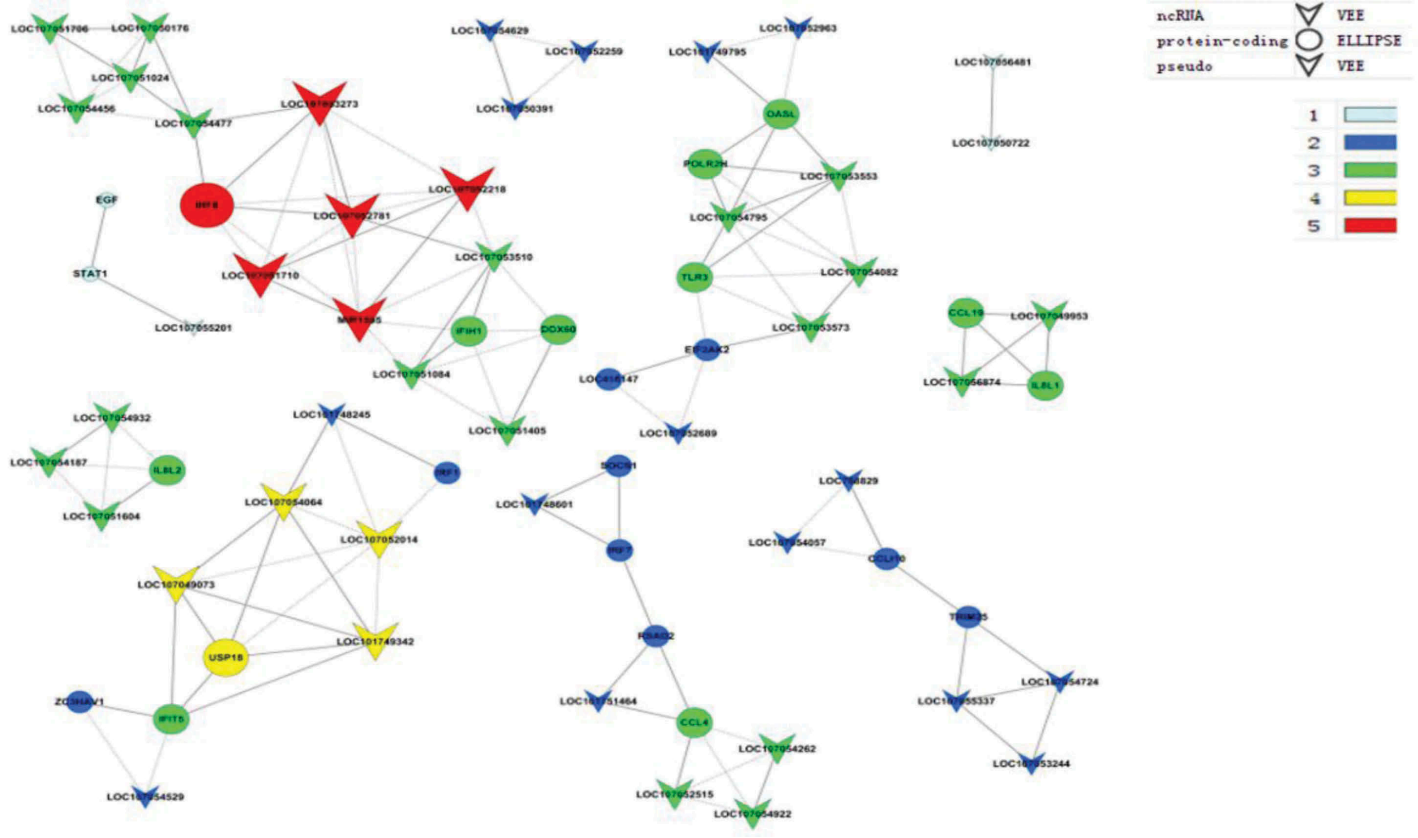
LncRNA-mRNA co-expression networks. A dot represents a differentially expressed mRNA, and a triangle represents a differentially expressed lncRNA. Genes in the same k-core are indicated with the same color. The progression from gray to red represents increasing k for the k-core. The color scale bar is shown.

To further examine the functions of the differentially expressed lncRNAs, lncRNA-mRNA co-expression networks were constructed. We first identified and selected the differentially expressed mRNAs and lncRNAs significantly enriched in GO terms and pathways related to the replication of IBDV. We then constructed and analyzed 10 lncRNA-mRNA co-expression networks (Figure 5). The lncRNA-mRNA gene pair loc107051710-*IRF8* had the best correlation.

### Validation of RNA-seq data by Rt-qPCR

**Figure 6.**
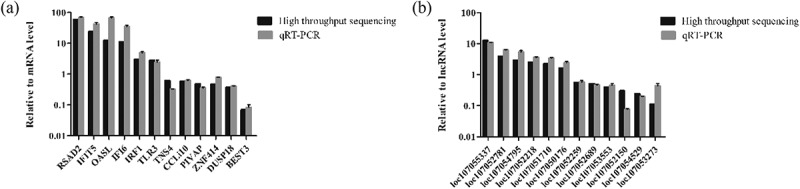
Validation of differentially expressed mRNAs and lncRNAs by RT-qPCR. (a) Transcript levels of mRNAs. (b) Transcript levels of lncRNAs. RT-qPCR experiments were performed in triplicate.

To confirm the accuracy of RNA-seq, several differentially expressed mRNAs and lncRNAs were selected and analyzed by RT-qPCR. The RT-qPCR results were consistent with those from RNA-seq (Figure 6(a,b)), indicating the validity of the RNA-seq data.

### Effect of silencing lncRNA loc107051710 on IBDV replication

**Figure 7.**
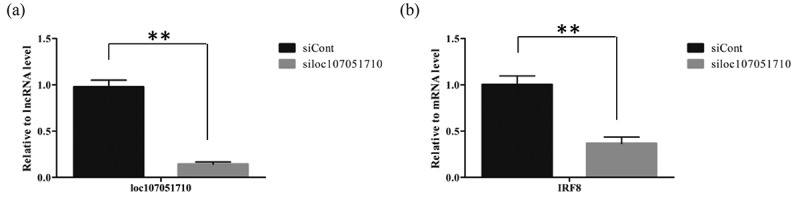
Effect of silencing loc107051710 on *IRF8* expression. DF-1 cells were transfected with either the siControl or siloc107051710 and then infected with IBDV (MOI = 1) for 24 h. The expression levels of loc107051710 and *IRF8* were analyzed by RT-qPCR. RT-qPCR experiments were performed in triplicate. Significant differences between the treated and control groups are indicated as **P < 0.01.

**Figure 8.**
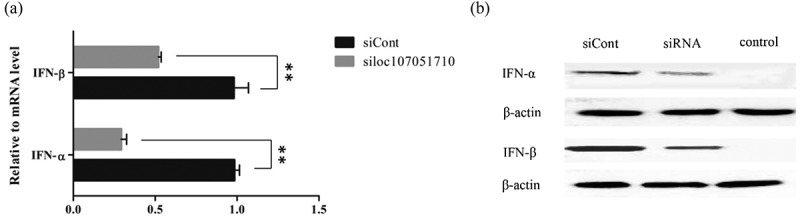
Effect of loc107051710 silencing on IFN-α and IFN-β production. DF-1 cells were transfected with either the siControl or siloc107051710 and then infected with IBDV (MOI = 1) for 24 h. The expression levels of IFN-α and IFN-β were measured by RT-qPCR and western blotting. (a) Changes in IFN-α and IFN-β mRNA levels. (b) Changes in IFN-α and IFN-β protein levels. RT-qPCR experiments were performed in triplicate. Significant differences between the treated and control groups are indicated as **P < 0.01.

**Figure 9.**
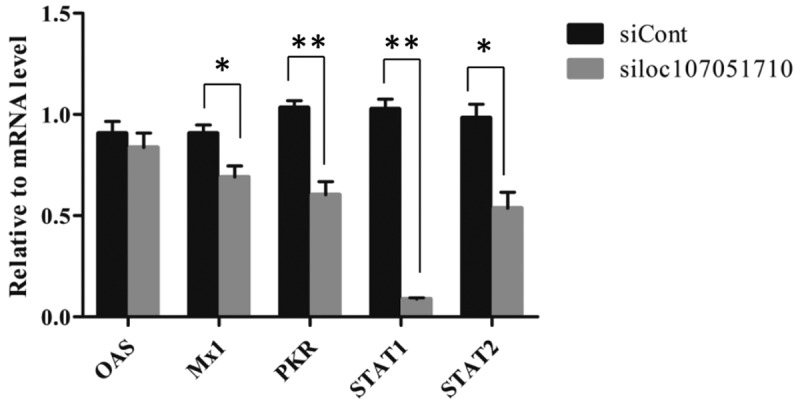
Effect of loc107051710 silencing on ISGs and STATs transcription. DF-1 cells were transfected with either the siControl or siloc107051710 and then infected with IBDV (MOI = 1) for 24 h. The expression levels of ISGs and STATs were measured by RT-qPCR. The experiments were performed at least in triplicate. Significant differences between the treated and control groups are indicated as *P < 0.05 and **P < 0.01, respectively.

**Figure 10.**
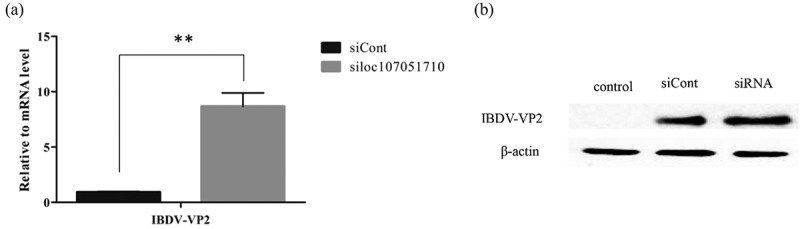
Effect of silencing loc107051710 silencing on IBDV VP2 production. DF-1 cells were transfected with siControl or siloc107051710 and then infected with IBDV (MOI = 1) for 24 h. The level of IBDV VP2 expression was measured by RT-qPCR and western blotting. (a) Changes in IBDV VP2 mRNA levels. (b) Changes in IBDV VP2 protein levels. RT-qPCR experiments were performed in triplicate. Significant differences between the treated and control groups are indicated by **P < 0.01.

To further illustrate the association of the loc107051710 with *IRF8*, the lncRNA was knocked-down in DF-1 cells using a specific siRNA. The siRNA for loc107051710 was effective in reducing the expression of the lncRNA (Figure 7(a)). Moreover, the decrease in loc107051710 expression was accompanied by a decrease in *IRF8* expression (Figure 7(b)). To determine whether the reduction in loc107051710 expression resulted in the reduction of type I IFNs (IFN-α and IFN-β), STATs (STAT1 and STAT2), and ISGs (OAS, Mx1, and PKR) expression, their expression levels were measured by RT-qPCR. Surprisingly, silencing loc107051710 decreased the levels of type I IFNs, STATs, and ISGs (Figures 8 and 9). To verify the antiviral effect of loc107051710, we infected siControl or siloc107051710-transduced DF-1 cells and measured the expression of IBDV. The results revealed that IBDV replication was increased by loc107051710 knockdown (Figure 10).

### Determination of location of loc107051710 in DF-1 cells by application of fluorescence in situ hybridization

**Figure 11.**
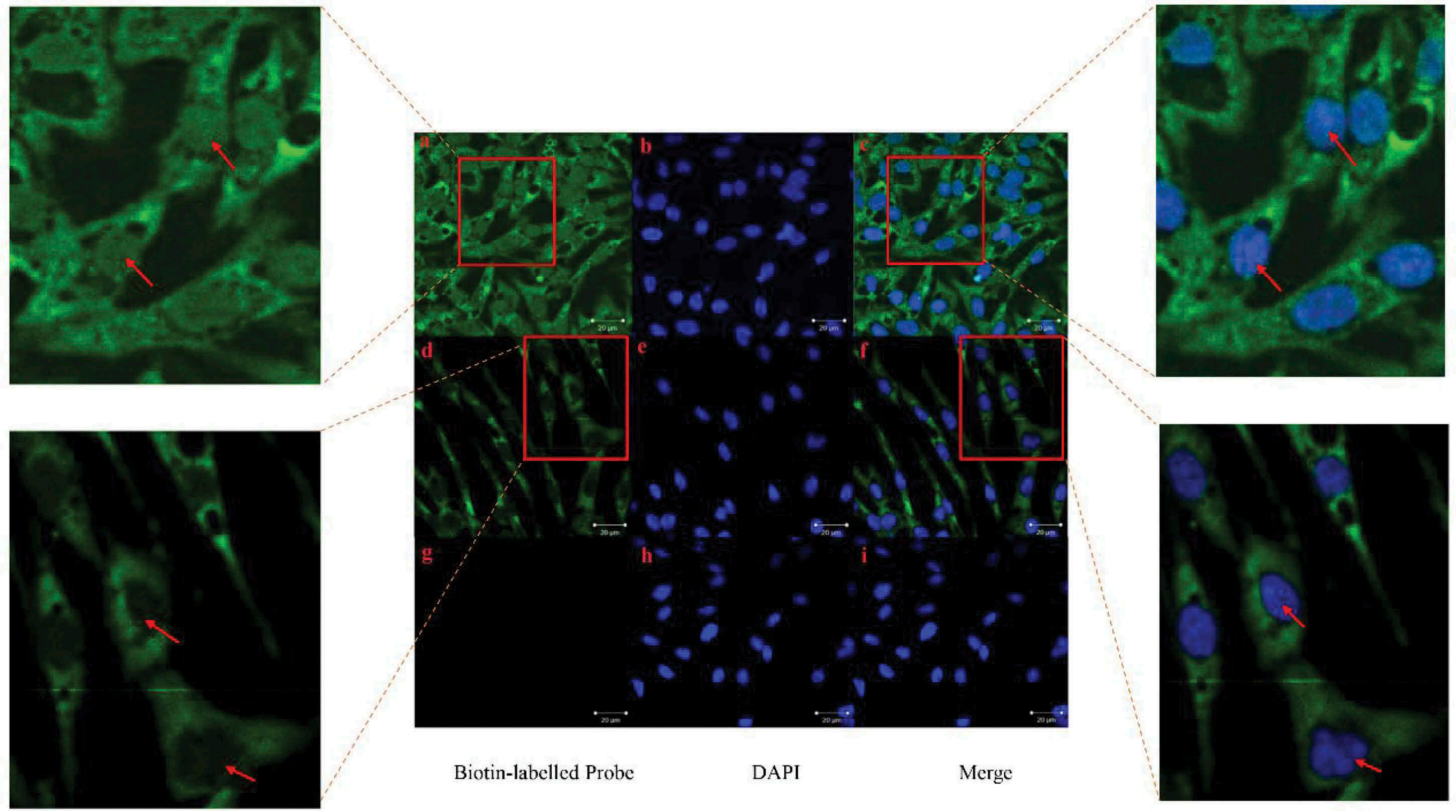
RNA FISH analysis of loc107051710 localization in IBDV-infected DF-1 cells 12 h post infection. (a–c) Non-infected DF-1 cells with antisense loc107051710 probe; (d–f) IBDV-infected DF-1 cells with antisense loc107051710 probe; (g–i) IBDV-infected DF-1 cells with sense loc107051710 probe. The nucleus is stained blue with DAPI. Green indicates the location of the loc107051710 probes.

Using FISH, loc107051710 was detected in both the cytoplasm and nucleus of uninfected DF-1 cells (Figure 11(a–c)). However, it was mainly detected in the cytoplasm after infection with IBDV (Figure 11 d-f), and the results showed that Loc107051710 did not bind to the sense probe in IBDV-infected DF-1 cells (g-i).

## Discussion

IRFs are a family of transcription factors that were originally isolated as positive and negative regulators of IFNs and IFN-responsive genes [34]. More recently, extensive studies of the IRF family have confirmed that IRFs have gained great attention due to their diverse roles such as in the initiation of antiviral responses, regulation of inflammatory cytokine expression, and control of cell cycle and apoptosis [35,36]. Numerous pattern recognition receptor that can detect cytosolic nucleic acids have been identified and most of them can evoke type I IFN responses via activation of IRFs [37]. The biological activities of type I IFN are initiated by binding to the type I IFN receptor. This leads to the activation of the receptor associated tyrosine kinases JAK1 and Tyk2, which phosphorylate STAT1 and STAT2. Phosphorylated STAT1 and STAT2 interact strongly with DNA-binding protein IRF-9. The IRF-9-STAT1-STAT2 heterotrimer is called ISGF3, which participates in the induction of the expression of ISGs, including OAS, Mx1 and PKP [38]. These ISGs can directly inhibit the replication, assembly and proliferation of the virus. To date, eight IRF homologues have been identified in chickens, though their functions are not yet well defined [39]. *IRF8* (also known as IFN consensus sequence-binding protein [ICSBP]), a member of the IRF family, is expressed by B cells, dendritic cells (DCs) and macrophages, and has a role in the regulation of innate immune responses [40,41]. As an important transcription mediator, *IRF8* is also required for type I IFN induction in virus-stimulated DCs [42]. It appears that *IRF8* is involved in the transcriptional regulation of type I IFN genes; *IRF8* can bind to promoters of IFN-α/β genes to promote the transcription of IFN-α/β [43]. Accordingly, it plays a critical role in the initiation of host innate immune responses to viral infection.

It is well known that host cells can regulate the expression levels of various mRNAs during viral infection. In the current study, differentially expressed mRNAs were analyzed by RNA-seq in DF-1 cells after infection with IBDV. Many of these mRNAs were associated with the antiviral responses of DF-1 cells. Previous studies have demonstrated that IBDV infections induce the expression of different cytokine genes [18]. Furthermore, the genes of cytokines that initiate inflammatory responses, including the *IL8*, nitric oxide synthase, and cyclooxygenase-2 genes, were reported to be upregulated [44,45]. In the present study, we similarly found that the expression of *STAT1, IL8L1*, and *IL8L2* was increased in DF-1 cells after infection with IBDV. Regulatory factors that could modulate the expression levels of these cytokine genes were also over-expressed, including IFN-induced GTP-binding protein Mx1 and IFN-induced protein with tetratricopeptide (IFIT). *IFIT5* is a member of the IFIT family, which can be triggered by viral infection [46] and enhances the antiviral response by promoting IRF3- and NF-κB-mediated gene expression [47]. Our results showed that *IFIT5* expression was 23.8-fold higher and that expression of the NF-κB-mediated genes *IL8L1* and *IL8L2* were 1.58-fold and 1.52-fold higher, respectively, in the infected group than in the control groups.

Radical S-adenosyl methionine (RSAD2) is an endoplasmic reticulum-associated virus inhibitory protein that can be induced by double-stranded RNA viral infection [48], as well as in an IFN-independent pathway by *IRF1* [49]. In our study, *IRF1* and *RSAD2* expression were 3.01-fold and 58.4-fold higher, respectively. Although the specific antiviral mechanism of *RSAD2* has not yet been determined, it is thought to indirectly inhibit viral replication by regulating cell survival [50]. Moreover, the upregulation of the tripartite motif-containing 25 (TRIM25) protein at the beginning of the infection may be associated with RIG-I in the detection of viral RNA intermediates [51]. A previous study has demonstrated that RIG-I can upregulate type I IFNs and reduce viral gene expression [52]. Our results showed that *TRIM25* expression was increased 1.6-fold, and the RIG-I-like receptor signaling pathway was significantly enriched during infection. The differential expression patterns of these mRNAs were consistent with those reported in previous studies. We believe that these over-expressed mRNAs may enhance the antiviral ability of DF-1 cells during the early stage of IBDV infection.

In this study, the type I IFN signaling pathway showed the highest enrichment. Type I IFNs can up-regulate the expression of hundreds of ISGs by activating the well-characterized JAK-STAT pathway [53]. Several studies have shown that ISGs induced by type I IFNs contribute to the antiviral response [54,55]. To determine whether differentially expressed lncRNAs play an antiviral role in IBDV-infected DF-1 cells by regulating interferon expression, we first screened out differentially expressed mRNAs significantly enriched in the GO terms and KEGG signaling pathways with antiviral activity. Next, we selected all the differentially expressed lncRNAs and mRNAs enriched in the GO terms and KEGG pathways to construct 10 lncRNA-mRNA co-expression networks. While there were many regulatory relationships in the networks, loc107051710, loc107052218, loc107052243, and loc107052781 had a close relationship with the *IRF8* gene, of which loc107051710 and *IRF8* gene pair had the closest relationship.

We confirmed the cascade relationship between loc107051710 and *IRF8* and found that loc107051710 positively regulates the expression of *IRF8*. In addition, we found that loc107051710 acts as a positive transcriptional regulator of the antiviral-related IFN-α, IFN-β, STAT1, STAT2, OAS, Mx1 and PKR. Therefore, we speculate that loc107051710 promotes the production of IFN-α and IFN-β by regulating *IRF8*, thereby promoting ISGs antiviral activity. However, whether loc107051710 directly regulates *IRF8* expression remains unknown.

The loc107051710 seems to possess antiviral function. However, it was unclear where loc107051710 was localized in uninfected cells and where it exerted antiviral effects after DF-1 cells were infected with IBDV. FISH showed that the amount of loc107051710 increased and shifted from the nucleus to cytoplasm during infection, indicating that it plays an antiviral role not only at the transcriptional level, but also at the post-transcriptional level. Therefore, the antiviral potential of the loc107051710 may be greater than the other lncRNAs analyzed in this study.

In conclusion, we profiled lncRNAs and mRNAs from IBDV-infected DF-1 cells, and based on GO and KEGG pathway enrichment analyses, we identified the differentially expressed mRNAs with antiviral functions. Notably, lncRNA-mRNA co-expression analysis revealed the potential antiviral function of the loc107051710, which involved the regulation of type I IFN, STAT, and ISG production to prevent IBDV infection. In brief, it was concluded that down-regulation of the long non-coding RNA loc107051710 enhanced the replication of infectious bursal disease virus by reducing interferon production. Vice versa up-regulation of the long non-coding RNA loc107051710 suppressed the replication of infectious bursal disease virus by elevated interferon production. Our findings provide a valuable foundation for future studies of the molecular mechanisms of innate anti-IBDV responses in DF-1 cells.
